# 

**Published:** 1845-04

**Authors:** 


### BOOKS RECEIVED FOR REVIEW.

1. The Natural History of Animals. By Th. Rymer Jones, F.R.S. Vol. I. 8vo, pp. 362.—London, 1845. 12s.

2. Modern Cookery in all its branches. By Eliza Acton, 1845. 8vo, pp. 684.

3. The General Nature and Treatment of Tumours. By S. Macilwain, Surgeon. — London, 1845. 8vo, pp. 220. 5s.

4. Address at the Anniversary Meeting of the Entomological Society. By George Newport, President.—London, 1845. 8vo.

5. Human Health, or the Elements of Hygiene. By R. Dunglison, M.D. &c.—Philadelphia, 1844. 8vo, pp. 464.

6. Practical Observations and Suggestions in Medicine. By Marshall Hall, M.D. F.R.S. &c.—London, 1845. 8vo, pp. 360. 8s. 6d.

7. The Enquirer, devoted to free discussion as to the proper use of Alcoholic Poisons.—Albany, 1842-3. 4to.

8. Recherches de Pathologie Comparée. Par Dr. Ch. F. Heusinger.—Cassel, 1844. 4to, pp. 63, and DXLIX.

9. A Treatise on Corns, Bunions, &c. By L. Durlacher, Chiropodist to the Queen.—Lond. 1845. 8vo, pp. 196. 10. 6d.

10. The Structure of the Lungs, as illustrative of the power and wisdom of God. By John Moore.—Birmingham, 1845. 8vo, pp. 106.

11. An Address to the Middle and Working Classes on the causes and prevention of Diseases. By W. Strange, M.D.—Ashton, 1845. 8vo, pp. 68.

12. Sopra Gentile Da Fuligno medico illustre del Secolo XIV. Del Dottor G. Girolami.—Napoli, 1844. 8vo, pp. 59.

13. Medical Education. A Lecture delivered at King's College. By J. F. Royle, M.D. F.R.S.—London, 1845. 12mo, pp. 64. 2s.

14. An Experimental Inquiry into the Pathology and Treatment of Asphyxia. By J. E. Erichsen.—London, 1845. 8vo, pp. 52.

15. Deux Cas d'Albuminurie chez le Cheval. Par S. Verhegen.—Bruxelles, 1843. 8vo, pp. 32.

16. A Practical Treatise on Midwifery. By F. J. Moreau. Translated from the French by T. F. Bellow, M.D.; and Edited by P. B. Goddard, A.M. M.D. With Eighty Plates.—Philadelphia, 1844. 4to, pp. 228.

17. Die Herzkrankheiten. Von F. Zehetmayer, M.D. &c.—Wien, 1845. 8vo, pp. 412.

18. A Treatise on Operative Surgery. With Eighty Plates, containing 486 Illustrations. By J. Pancoast, M.D. &c.—Philadelphia, 1844. 4to, pp. 380.

19. An Introductory Lecture on Medical Education. By Charles Lee, M.D.—Geneva (America), 1844. 8vo.

20. A collection of Cases of Apoplexy; with an Explanatory Introduction. By E. Copeman, Surgeon.—London, 1845. 8vo, pp. 206. 7s.

21. The Diagnosis, Prevention, and Treatment of Diseases of the Heart and of Aneurism. By J. J. Furnivall, M.D.—London, 1845. 8vo, pp. 216. 7s.

22. The Anatomy of Sleep. Second Edition. By E. Binns, M.D.—London, 1845. 8vo, pp. 506. 10s. 6d.

23. Londres et les Anglais des Temps Modernes. Par le Dr. Bureaud-Rioffrey. — Londres, 1844. 8vo, pp. 430.

24. The Medical Student; or Aids to the Study of Medicine. By R. Dunglison, M.D. &c. — Philadelphia, 1844. 8vo, pp. 312.

25. Second Report of the Sanitary Commission. —London, 1845. Folio.

26. Causes générales des Maladies Chroniques, specialement de la Phthisie Pulmonaire. Par A. Fourcault, M.D.—Paris, 1844. 8vo, pp. 480.

27. The Chemistry of Vegetable and Animal Physiology. By Dr. G. J. Mülder. Translated from the Dutch by Dr. P. F. H. Fromberg; with an Introduction and Notes by J. F. W. Johnston, F.R.SS. L. & E. Pt. I.—Edinb. 1845. 8vo, pp. 184. 4s.

28. Transactions of the Medical and Physical Society of Bombay. Nos. V-VI. 1842-3. 8vo, pp. 196, 250.

29. The History of the Rabbis, &c.—Lond, 1845.

30. An Essay on Madness, containing the Outlines of a new Theory. By R. Spear.—Toronto, 1844. 8vo, pp. 25.

31. The Pharmacopœia a Dead Letter. By W. Bastick.—London, 1845. 8vo, pp. 15. 1s.

32. De Tenotomia talipedibus applicata commentatio quam scripsit Chr. Weis, L.M.—Hafniæ, 1844. 12mo, pp. 94.

33. Medicina Gymnastica. By C. Ehrenhoff. —London, 1845. 8vo, pp. 18.

34. An Explanation of the real process of "Spontaneous Evolution." By T. C. Douglas, M.D.—Dublin, 1844.

35. Practical Remarks on some Exhausting Diseases, particularly incident to Women. By Sir James Eyre, M.D. — London, 1845. 8vo, pp, 75. 4s.

36. Practical Observations on the Diseases most fatal to Children. By P. Hood. London, 1845. 8vo, 231. 6s.

LIBRARY OF THE

Orleans Parish Medical Society

### INDEX TO VOL. XIX

OF THE

### BRITISH AND FOREIGN MEDICAL REVIEW.

| | PAGE |
|---|---|
| Abercrombie, Dr. obituary of | 312 |
| Abscesses, intra-pelvian | 559 |
| Absorption, physiology of | 278 |
| Ackermann, Dr. biography of | 535 |
| Air, on pure and impure | 2 |
| purification of | 14 |
| Akesios, Professor Marx's | 121 |
| Alum, a remedy in abdominal typhus | 366 |
| Anatomy, Lizars' elements of | 243 |
| report of the progress of | 249, 563 |
| Aneurism, cure of by compression | 525 |
| Animal magnetism, treatises on | 428 |
| Angina pectoris, treatment of | 25 |
| Animal heat, our present knowledge of | 266 |
| Ansted, Professor, his geology | 554 |
| Aorta, anatomy of | 487 |
| Army, on the medical hygiene of | 377 |
| Arnold, M. on the spinal nerves | 558 |
| Arsenic, as a poison and remedy | 375 |
| Arteries, Professor Quain's treatise on | 486 |
| development of | 590 |
| Asthma, capriciousness of | 24 |
| Ashwell, Dr. on the diseases of women | 344 |
| Asylums, lunatic, of Paris, account of | 319 |
| private, at Paris | 608 |
| Atmosphere, artificial | 15 |
| | |
| Barracks, on the site and construction of | 383 |
| Bell, Mr. on the cure of phthisis | 140 |
| Bennett, Dr. his treatise on inflammation | 244 |
| Bicêtre, account of | 292 |
| Biography, medical | 530 |
| Birth of man, aphorisms on | 557 |
| Bischoff on menstruation | 84 |
| Blood, on the moving powers of | 557 |
| our present knowledge of | 252 |
| Books received for review | 316, 618 |
| Brain, on the reflex function of | 298 |
| recent anatomy of | 578 |
| Bronchitis, diagnosis of | 110 |
| Brachial artery, anatomy of | 499 |
| ligature of | 501 |
| Brussels, on the mortality of | 126 |
| Bunions and corns, treatise on | 560 |
| Burns, operations on cicatrices from | 410 |
| | |
| Cancer of the stomach, latent | 27 |
| Carcinoma, uterine, treatment of | 352 |
| Carpenter, Dr. his principles of physiology | 245 |
| Chapman, Dr. on the diseases of the viscera | 21 |
| Chailly, M. on midwifery | 416 |
| Charenton, Dr. Conolly's account of | 594 |
| Charter of the College of Physicians | 553 |
| new, for general practitioners | 614 |
| Cheek, plastic operations on | 403 |
| Cities, great, mortality of | 126 |
| Chemical composition of the body | 249 |
| Chemistry, manual of | 245 |
| on pathological | 364 |
| Child, relation of its sex to parturition | 541 |
| Chlorosis, treatment of | 345 |
| Cicatrices from burns, operations for | 410 |
| Circulation, present state of our knowledge of | 260 |
| Clairvoyance, pretended, cases of | 453 |
| Clothing, on military | 384 |
| Cod-liver oil in phthisis | 141 |
| College of Physicians, new charter of | 553 |
| Conolly, Dr. on the lunatic asylums of Paris | 281 |
| Conolly, Dr. on the Parisian asylums | 594 |
| Consciousness, double, cases of | 444 |
| Constipation, Dr. Chapman on | 31 |
| Contagion, on the matter of | 363 |
| Copulation, anatomy of the organs of | 507 |
| Corns and bunions, treatise on | 560 |
| Cranium, morbid growth of, in puerperal women | 393 |
| Creation, vestiges of the natural history of | 155 |

PAGE

Cretinism, Dr. Knolz on . 372
Cronin, Mr. Dr. his books . 242

Diarrhea, American treatment of . 30
Dictionary, biographical . . 530
Diet of soldiers . . . 385
Digestion, chemistry of . . 268
Diseases of the thorax, Dr. Chapman on 22
Dispensaries, Indian, charitable . 72
Ducpetiaux, on the mortality of Brussels 126
Durlacher, Mr. his treatise on corns 560
Dyspepsia, causes of, in America . 27
Dystocia, Dr. Naegele on . 60
Dypsychia . . . 443

Electricity, as a remedy . . 368
Embryo, physiology of . . 587
respiration of . . 592
Emphysema, diagnosis of . 107
Ethics of medicine . . 121
Evils of large cities, and their cure . 126
Excretion, report on the function of 566
Eye, anatomy and physiology of . 584
Eyelids, anatomy of . . 585
plastic operations on . 409

Femoral artery, anatomy of . 502
ligature of . 504
Fergusson, Mr. his new operation . 415
Foville, M. on insanity . . 596

Gangrena senilis, treatment of . 528
Gastritis, Dr. Chapman on . 26
Gastric fluid, properties of . . 270
Gastrodynia, cured by milk . 29
General practitioners, association of 614
Genital organs, anatomy of . 586
Geology, Professor Ansted's . 554
Glands, vascular, report on . 570
Graham, Sir James's new medical bill 193
Grantham, Mr. his facts in medicine 247
Grassi, Dr. on the plague . 41
Grohmann, Dr. on the plague . 41
Guyot, M. on heat in ulcers, &c. . 182

Habits, influence of on health . 56
Halford, Sir Henry, obituary of . 315
Hall, Dr. M. his practical observations 561
Heart, normal and anormal site of . 117
Heat, employment of in ulcers, &c. 182
Hoblyn's dictionary of medical terms 245
Hematemesis complicating chlorosis 345
Henle and Kölliker on the pacinian corpuscles . . 78
Hereditariness, Dr. Levy on . 55
Hocken, Dr. on the cure of phthisis 140
Hoffbauer on the law in insanity . 33

PAGE

Holland, Dr. on the moving powers of the blood . . 557

Houses, on warming and ventilating . . . 1

Hydrometra, case of . . 355

Hygiene, Dr. Levy on . . 51

military . . 377

Hysteric mesmerism . . 432

Imminence, morbid . . 57
Indian medicine and surgery . 72
Infants, on the pulse of . . 390
Inflammation, Dr. Bennett's treatise on . . . 244
Inflammation, treatment of by mercury . . . 527
Insane, classification of . . 330
Insanity, on the legal relations of . 33
Inversion of the uterus . 356, 423
Iodine, new mode of treating syphilis with . . . 516

Jameson, Mr. on vivisection . 248
Jones, Professor, his natural history 556

Kobelt, M. on the organs of copulation . . . 501
Kidneys and their secretion, report on 569

Labour, different stages of . 63
Lacrymal gland, anatomy of . 588
Laycock, Dr. on the function of the brain . . . 298
Lefevre, Sir George, his apology for the nerves . . . . 240
Levy, Dr. on public and private hygiene . . . . 51
Leucorrhea, treatment of . . 347
Lips, plastic operations on . 404
Liver, anatomy and physiology of . 274
Lizars, Dr. his elements of anatomy 243
Lugol, M. on scrofula . . 239
Lunacy, report of commissioners on 317

Mayer, Dr. on the circumvolution of the cord . . . 546
Mayo, Dr. on the impunity of the insane . . . 33
Medical aphorisms and forms . 125
Medical bill, new, for Norway . 188
Medical reform, works on . 193
Memoirs of the society of observation 390
Menstruation, theories of . 84
Mercury in phthisis . . 145
its use in inflammation . 527
Mesmerism, treatises on . . 428
Midwifery, Dr. Naegele on . 58

PAGE
Midwifery, treatises on practical . 416
Military medical guide books . 377
Miller, Professor, his Principles of Surgery . . . 524
Moj'sisovics, Dr. on the cure of syphilis by iodine . . 516
Mouth, plastic operations on . 402
Moreau, M. on midwifery . . 416
Mütter, Dr. on plastic operations . 396
Müller, Professor, on osteoid tumours 537

Naegele, Dr. on midwifery . . 58
Naphtha, use of in phthisis . . 146
Natural history of animals . . 556
Nerve, anatomy and physiology of the olfactory . . . 579
optic . . . ib.
fifth . . . ib.
auditory . . . ib.
facial . . . 580
glosso-pharyngeal . . 580
pneumo-gastric . . 581
Nerves, anatomy and physiology of 579
apology for . . . 240
new corpuscles of . . 78
peripheral terminations of . 574
Neuroses, memoir on . . 555
Nervous system, report on . . 572
development of . 592
general physiology of 575
centres, anatomy and physiology of . . 577
New Medical Bill, second edition . 549
Norway, new medical bill for . 188
Nose, plastic operations on the . 399
Nutrition, report on . . 563

Obstetric Medicine, Dr. Ramsbotham on . . . . . 235
Operations in the mesmeric state . 434
Organs, internal, on the site of . 103
Os uteri, occlusion of . . 353
Ova, periodical maturation of . 84
Ovum, physiology of . . 587
Oxygen, or vital air, remedial use of 233

Pacinian corpuscles of the nerves . 78
Paget, Mr. his report on anatomy 249, 563
Palate, cleft, new operation for . 415
Palate, Dr. Mütter on cleft . . 408
Palate, operation for fissure of . 412
Paris, account of the asylums of . 281
Pauper lunatics, asylums for . 319
Pelvic abscesses, treatise on . . 557
Pereyra, M. on the cure of phthisis 140
Phthisis, Dr. Chapman on . . 22
independent of scrofula . 22
Phthisis, physical signs of . . 113

PAGE
Phthisis, on the curability of . 140
treatises on the cure of . 140
Physiology, report of the progress of 249
Physicians under the letter A. . 533
Physiology, Dr. Carpenter's principles of . . . 245
Plague, on its contagion . . 41
Plastic surgery, treatises on . 396
Pleurisy, physical signs of . . 111
Pneumonia, diagnosis of . . 110
Prescriber's pharmacopœia . . 246
Pressure, cure of aneurism by . 525
Principles of surgery, Professor Müller's . . . . 524
Puberty, on the period of . . 84
Pulse of infants, M. Valleix on . 390

Quain, Mr. his treatise on the arteries . . . . 486
Quarantine, works on . . . 41

Raciborski, on menstruation . 84
Ramsbotham, Dr. on obstetric medicine . . . . 235
Ranking, Dr. his translation of Lugol 239
Ray on the jurisprudence of insanity 33
Riadore, Dr. his new book . 233
Reflex function of the brain . 298
Reform, medical, essay on . . 193
Reid, Dr. on warming and ventilation 1
Religious services for the insane . 339
Remedies, American, odd . . 29
Remembrances, medical . . 246
Respiration, present state of our knowledge of . . . 263
Restraint or non-restraint in insanity 334
Retroversion of the uterus . . 422
Rouen, lunatic asylum of . . 603

Salpêtrière, account of . . 281
Schools for the sons of medical men 613
Serre, M. on deformities of the face 396
Sex of the child, its relation to parturition . . . . 541
Shock from injury, treatment of . 529
Sibson, Mr. on the site of the internal organs . . . 103
Simpson, Professor, on the sex of the child . . . 541
Skeleton, report on . . . 571
Sleep-waking . . . 440
Smyth, Dr. his medical contributions 237
Society of medical observation . 390
Soldiers, on preserving the health of 377
Somnambulism, spontaneous . 440
Spinal irritation, Dr. Türck on . 96
phenomena of . 97
Spinal nerves, on the functions of . 558

PAGE

Spinal cord, anatomy and physiology of . . . . 575

Spine, treatment of curvature of . 370

Spleen, diseases of . . . 31

Stomach, anatomy and physiology of 269

Subclavian arteries, anatomy of . 490

ligature of . 497

Surgery, plastic, treatises on . . 396

the principles of . . 524

Syphilis, new mode of cure of, by iodine . . . . 516

Taylor, Mr. his thermometrical table 562

Temperaments, Dr. Levy on . . 53

Temperatures, table of . . 562

Theory, influence of, on medicine . 361

Thermometers, new scale of . . 562

Tissues, anatomy and physiology of 256

Tongue, anatomy of . . 586

Trance, hysteric . . . 437

Tumours, osteoid . . . 537

Türck on spinal irritation . . 69

Turner, Mr. his manual of chemistry . . . 245

PAGE

Typhus, abdominal, of Vienna . 366

Umbilical cord, danger of its circumvolutions . . . . 546
Uterus, on irritable . . 348
malignant tumours of . 349

Vade mecum, anatomist's . . 243
Ventilation, illustrations of . . 1
Vestiges of the natural history of creation . . . 155
Vienna, transactions of medical society of . . . 360
Vivisection, remarks on . . 248
Voice, report on . . . 572

Wales, state of lunatics in . . 342
Warming, Dr. Reid on . . 1
Wilson, Mr. his anatomist's vade mecum . . .
Witchcraft, history of . 470
Women, treatise on the diseases of . 344

END OF VOL. XIX.

C. AND J. ADLARD, PRINTERS, BARTHOLOMEW CLOSE.

